# A Systematic Review of the Development and Validation of the Heat Vulnerability Index: Major Factors, Methods, and Spatial Units

**DOI:** 10.1007/s40641-021-00173-3

**Published:** 2021-04-27

**Authors:** Yanlin Niu, Zhichao Li, Yuan Gao, Xiaobo Liu, Lei Xu, Sotiris Vardoulakis, Yujuan Yue, Jun Wang, Qiyong Liu

**Affiliations:** 1 State Key Laboratory of Infectious Disease Prevention and Control, Collaborative Innovation Center for Diagnosis and Treatment of Infectious Diseases, National Institute for Communicable Disease Control and Prevention, Chinese Center for Disease Control and Prevention, 155 Changbai Road, Changping, Beijing, 102206 China; 2 Beijing Center for Disease Prevention and Control, Institute for Nutrition and Food Hygiene, Beijing, China; 3 Research Center for Preventive Medicine of Beijing, Beijing, China; 4 University College London, London, UK; 5 Key Laboratory of Land Surface Pattern and Simulation, Institute of Geographic Sciences and Natural Resources Research, Chinese Academy of Sciences, Beijing, China; 6 Ministry of Education Key Laboratory for Earth System Modeling, Department of Earth System Science, Tsinghua University, Beijing, China; 7 Center for Healthy Cities, Institute for China Sustainable Urbanization, Tsinghua University, Beijing, China; 8 National Centre for Epidemiology and Population Health, Research School of Population Health, Australian National University, Canberra, Australia

**Keywords:** Heat vulnerability index, Index development, Index validation, Influencing factors, Spatial units, Systematic review

## Abstract

**Purpose of review:**

This review aims to identify the key factors, methods, and spatial units used in the development and validation of the heat vulnerability index (HVI) and discuss the underlying limitations of the data and methods by evaluating the performance of the HVI.

**Recent findings:**

Thirteen studies characterizing the factors of the HVI development and relating the index with validation data were identified. Five types of factors (i.e., hazard exposure, demographic characteristics, socioeconomic conditions, built environment, and underlying health) of the HVI development were identified, and the top five were social cohesion, race, and/or ethnicity, landscape, age, and economic status. The principal component analysis/factor analysis (PCA/FA) was often used in index development, and four types of spatial units (i.e., census tracts, administrative area, postal code, grid) were used for establishing the relationship between factors and the HVI. Moreover, although most studies showed that a higher HVI was often associated with the increase in health risk, the strength of the relationship was weak.

**Summary:**

This review provides a retrospect of the major factors, methods, and spatial units used in development and validation of the HVI and helps to define the framework for future studies. In the future, more information on the hazard exposure, underlying health, governance, and protection awareness should be considered in the HVI development, and the duration and location of validation data should be strengthened to verify the reliability of HVI.

**Supplementary Information:**

The online version contains supplementary material available at 10.1007/s40641-021-00173-3.

## Introduction

In the context of climate change, the global mean surface air temperature has shown a rising trend over the last 100 years [1], which has led to an increase in the frequency, intensity, and duration of extremes of heat since 1950 [2]. The most immediate and direct impact of rising temperature on human health is the increase in heat-related mortality and morbidity worldwide [3–8]. The 2003 heatwave in Europe resulted in around 15,000 heat-related deaths in France and around 70,000 deaths across Europe [9]. The 2006 heat wave in CA, USA, resulted in around 1200 additional hospitalizations and 16,000 additional emergency room visits [10]. The 2010 heat wave in Russia caused an estimated 55,000 deaths [11]. Heat-related “adverse effects” go beyond deaths directly related to heat stress, also including heat stress-driven worsening of cardiovascular, diabetic, and respiratory conditions, as well as economic impacts caused by the loss of labor hours [12–14].

Vulnerability is broadly defined as the potential loss of property or life from environmental hazards [15]. The Intergovernmental Panel on Climate Change defines vulnerability as “the degree to which a system is susceptible to or unable to cope with adverse effects of climate change, including climate variability and extremes” [16]. Vulnerability is most often conceptualized as being constituted by components that include exposure and sensitivity to perturbations or external stresses and the capacity to adapt [17]. Exposure and sensitivity can be combined into a potential impact, which could further interact with adaptive capacity, resulting in vulnerability. Another common conceptualization is the hazards-of-place model of vulnerability utilized by Cutter et al. in the Social Vulnerability Index (SoVI) [18]. In this concept, the hazard potential is the result of interaction between risk (an objective measure of the likelihood of a hazard event occurring) and mitigation (measures to reduce risks or mitigate their impact), which can be moderated or enhanced by a geographic filter and the social fabric of the place. In addition, some researchers considered the engagement of stakeholders in the framework of vulnerability [19, 20]. Wilhelmi and Hayden [19] presented a new research framework for a multi-faceted, top-down and bottom-up analysis of local-level vulnerability to extreme heat, suggesting the integration of quantitative and qualitative data on social vulnerability and adaptive capacity that go beyond aggregate demographic information. The engagement of stakeholders as one of the inputs of quantitative and qualitative data could connect people and places to understand better local-level vulnerability and existing adaptation.

To date, with the profound understanding of vulnerability, numerous indices have been developed and applied in the heat vulnerability assessment based on various frameworks and objectives [21–24, 25•, 26], as following:

1. To identify vulnerable areas and populations at risk. For example, Wolf and McGregor developed an HVI for London, UK, in 2013 and found that vulnerability was higher in central London, including the areas in the central boroughs and in particular areas north of the Thames [27].
2. To assess the spatial and temporal distribution of vulnerability and to explain the reasons for these distributions. For example, Chow et al. developed the HVI maps in Metropolitan Phoenix, USA, between 1990 and 2000 to illustrate how climate, urban ecology, social status, and changing demography interact to create and change the spatial and temporal patterns of heat vulnerability [28].
3. To provide decision support with resource allocation in preparation for and response to heat-related events. For example, based on the HVI map in Pittsburgh, USA, Bradford et al. determined the optimal cooling center placement to address the vulnerability of at-risk populations, which is a cost-effective solution for city decision-makers [29].
4. To project future vulnerability. For example, using a HVI, Oh et al. assessed the health vulnerability to heat waves under the Representative Concentration Pathways (RCP) 8.5 scenario in the 2040s at the province level in South Korea and concluded that Daegu Metropolitan City was the most vulnerable region [30].

The HVI may meet multiple aims in a single study. However, it is often used by local public health practitioners and policymakers to identify the areas and population at risk for health protection. Vulnerability depends critically on context, and the factors that make a system vulnerable to heat will depend on the nature of the system [31]. Although the current HVIs are substantial, there is no reference for the generic determinants of vulnerability, which will be useful to undertake comparative assessments at the same level [17, 31]. In addition, researchers are faced with choices between plausible alternatives, thus bring their subjectivity into the modeling process [32]. Therefore, index validation via proper variables and methods plays a critical role in testing the HVI. However, few attempts have been made [33].

In this context, focusing on the development and validation of the HVI, the objective of this study is to identify the key factors, methods, and spatial units used in the development and validation of the HVI by summarizing and analyzing the existing literature and to discuss the underlying limitations of the used data and methods by evaluating the performance of the HVI.

## Methodology

In this study, the HVI is defined as a composition of the influencing factors of vulnerability that quantitatively reflects the vulnerability level of the population for high temperature or extreme heat events. The literature search was conducted to identify studies related to the development, application, and validation of HVI. We followed the guidelines of the Preferred Reporting Items for Systematic Reviews and Meta-Analyses (PRISMA) statements [34] to conduct the analyses. Several online databases including PubMed, Web of Science, Science Direct, China National Knowledge Infrastructure (CNKI), and Wanfang Data were queried. The research was limited to peer-reviewed journal articles published in English and Chinese between January 2010 and October 2020. The following keywords were used individually and in combination in the search phase: heat, heatwave(s), extreme heat, high temperature, thermal, vulnerability, risk, index (indices), assess, assessment, evaluation, and health. The detailed search strategy was attached in Supplementary Materials 1. Search results were then imported into EndNote where the duplicates were eliminated. We did not search for or include any unpublished studies or gray literature such as government or organization reports. The title and abstract screening and eligibility were conducted by two authors (i.e., YN and YG), and the full texts of the included articles were obtained for further assessment.

The inclusion criteria used in this study were as follows:

(1) An index that intended to quantify the vulnerability of population for heat or heatwave was developed and applied, especially in public health; (2) performance of the index was validated with the observed health data; (3) factors and methods used in the development and validation of the index were explicitly described; and (4) original research articles.

The exclusion criteria used in this study were as follows:

(1) Heat risk indices that have different definitions and purposes; (2) the index was targeted for certain populations (e.g., the elderly, children, outdoor workers), environment (e.g., indoor, workplace), disease (e.g., cardiovascular diseases, respiratory diseases), and realm (e.g., agriculture, aquaculture); (3) the index was applied for a projection of risk in the future; and (4) the index was targeted for climate change in broad terms or multiple hazards or coupled with air pollution.

The information concerning the data and methods used in HVI development and validation were then extracted to build the following three tables:

1. Basic information and geographical context: author, publication year, geographical region, geographical unit of spatial analysis, types of geographical unit (Table 1)
2. Index development: the factors for construction that were categorized into five aspects (hazard exposure, demographic characteristics, socioeconomic conditions, built environment, and underlying health) and developmental methods (Table 2)
3. Index validation: the factors for validation, time span and duration of used validation data, sample size, and validation methods (Table 3)

**Table 1:** Summary of the basic information and the geographical context in the studies

ID	Author	Publication year	Geographical region	Geographical unit of spatial analysis	Types of geographical unit
1	Johnson, Daniel P. et al.	2012	Chicago, Illinois, USA	Census block group	Census tracts
2	Reid, Colleen E. et al.	2012	California, Massachusetts, New Mexico, Oregon, Washington, USA	ZIP code	Postal code
3	Harlan, Sharon L. et al.	2013	Maricopa County, Arizona, USA	Census block group	Census tracts
4	Wolf, Tanja et al.	2013, 2014	London, UK	the Super Output Lower Level (SOA)	Census tracts
5	Maier, George et al.	2014	Georgia, USA	County	Administrative areas
6	Chuang, Wen-Ching et al.	2015	Phoenix, Arizona, USA	Census tract	Census tracts
7	Prudent, Natasha et al.	2016	Travis County, Texas, USA	Census block group	Census tracts
8	Kim, Do-Woo et al.	2017	Korea	County	Administrative areas
9	Krstic, Nikolas et al.	2017	Greater Vancouver, British Columbia, Canada	Postal code	Postal code
10	Nayak, S. G. et al.	2018	New York State, USA	Census tract	Census tracts
11	He, Cheng et al.	2019	Shanghai, China	Grid (500m)	Grid
12	Mallen, Evan et al.	2019	Dallas, Texas, USA	Census tract	Census tracts
13	Conlon, K. C. et al.	2020	Detroit, Michigan, USA	Census tract and block group	Census tracts

**Table 2:** Summary of the factor categories and methods used in the index development in the studies

ID	Categories					Total	Developmental method
	Hazard exposure	Demographic characteristics	Socioeconomic conditions	Built environment	Underlying health		
1	**√**	**√**	**√**	**√**		19	PCA
2		**√**	**√**	**√**	**√**	10	PCA
3		**√**	**√**	**√**		10	PCA
4		**√**	**√**	**√**	**√**	9	PCA
5		**√**	**√**	**√**	**√**	8	PCA
6		**√**	**√**	**√**	**√**	10	FA
7	**√**	**√**	**√**	**√**		7	PCA
8	**√**	**√**	**√**			4	Zero-inflated Poisson regression analysis
9	**√**	**√**	**√**	**√**		4	The summation model; the Heat Exposure Integrated Deprivation Index approach
10		**√**	**√**	**√**	**√**	13	PCA
11	**√**		**√**	**√**	**√**	7	FA
12		**√**	**√**	**√**	**√**	10	PCA
13		**√**	**√**	**√**		8	Unsupervised/supervised PCA

**Table 3:**
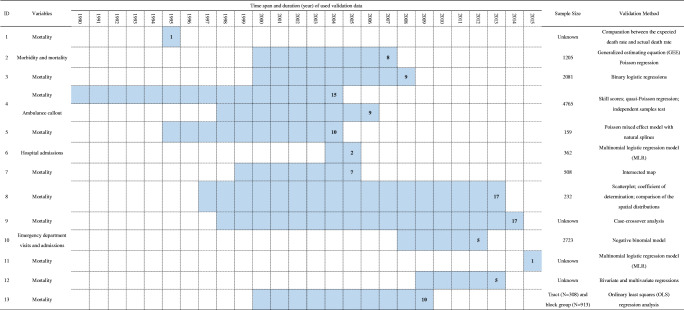
Summary of variables information and methods used in the index validation in the studies

## Results

### Overview of the Included Articles

**Fig. 1:**
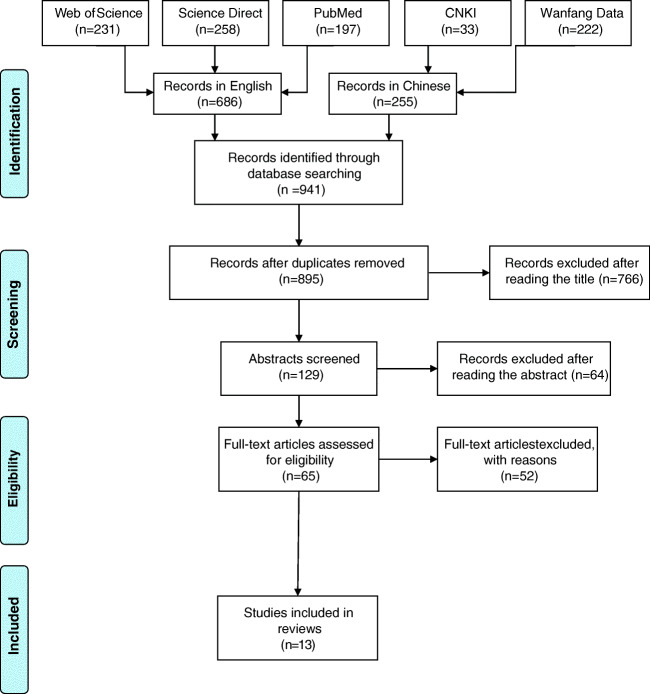
The literature selection process

Nine hundred and forty-one bibliographic records were identified through the initial search, including 686 records in English and 255 records in Chinese. After eliminating duplicates and screening titles, abstracts, and full texts, 13 studies were included in this review (Fig. 1). Particularly, Wolf et al. developed an HVI in 2013 to assess the intra-urban variability of vulnerability to heat wave events in London [33] and assessed it in 2014 [27]. Therefore, these two studies were merged. It should be noted that the total number of studies on the development and application of the HVI was 46 during 2010–2020. Among them, 13 studies involving the HVI validation with the observed health data were included in this review (Table 1). The list of the 33 excluded studies is attached in Supplementary Materials 2.

Among the 13 included studies, nine studies were carried out in the USA, and the other four studies were conducted in the UK, Canada, China, and Korea. Most studies (8 out of 13) used census-based unit in spatial analysis. A census block group (called the Super Output Lower Level (SOA) in the UK), that is, the smallest statistical unit in published census data, was used as the geographical unit of spatial analysis to provide a fine resolution in five studies. Other units used in the studies included census tract, postal code, and county. In addition, He et al. [35•] used a 500-m grid as the statistical basis for a study in Shanghai, China.

### HVI Development

**Fig. 2:**
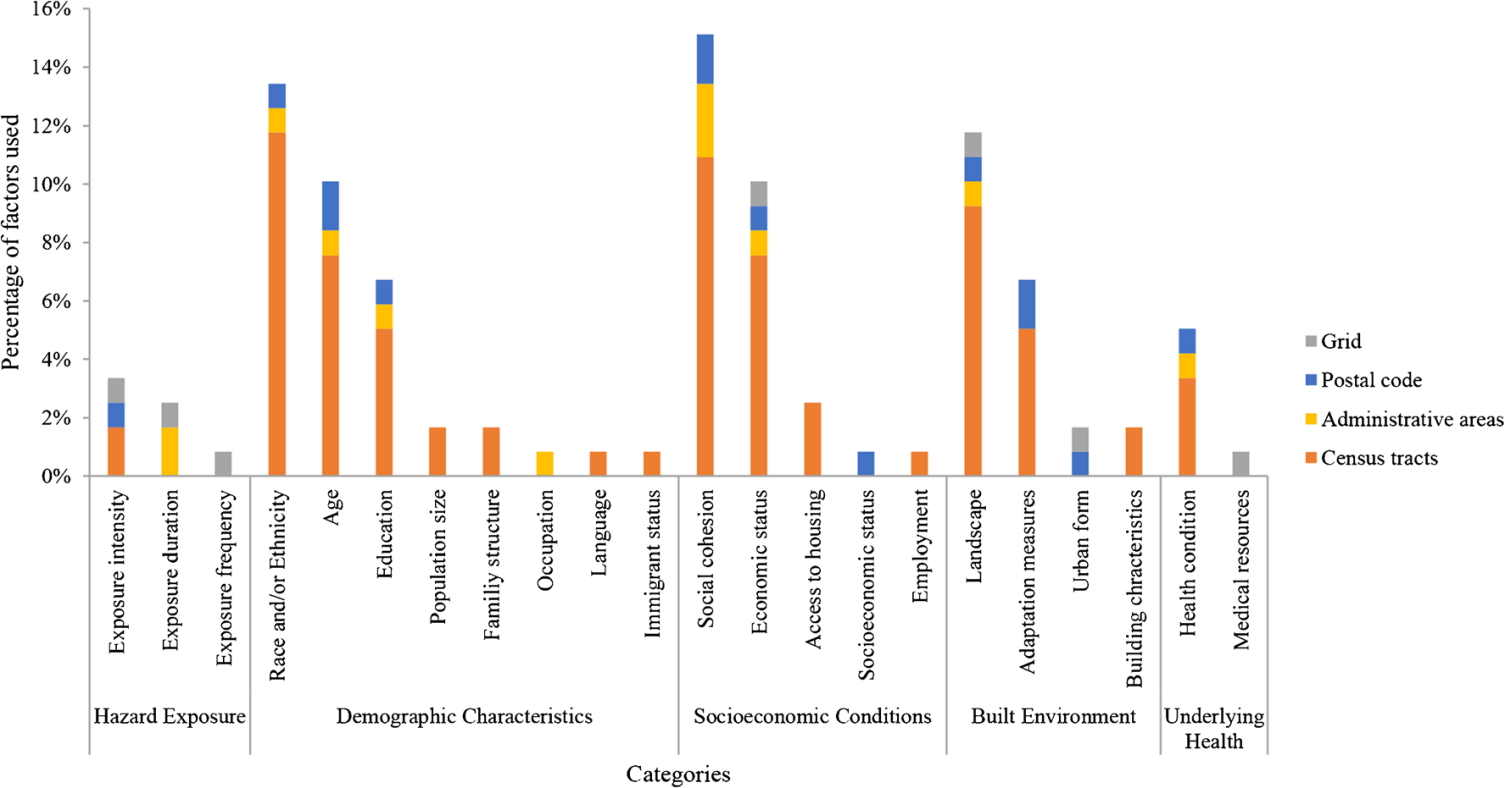
Summary of factors used in the index development by categories and types of geographical unit

The total number of factors used in the HVI development ranged from 4 to 19 (Table 2, a detailed inventory of factors is attached in Supplementary Materials 3). Almost all the studies used factors related to demographic characteristics, socioeconomic conditions, and built environment. More than 50% of studies (7 out of 13) incorporated health-related factors, while only five studies considered hazard exposure factors during index development. In detail, we analyzed the used factors by categories and types of geographical unit (Fig. 2). The top five factors used in all types were social cohesion (15%), race and/or ethnicity (13%), landscape (12%), age (10%), and economic status (10%). A similar distribution of the factors used in studies considered census tract as geographical unit was found. Nevertheless, exposure frequency, occupation, language, immigrant status, employment, and medical resources were seldom used, accounting for less than 1%. Principal component analysis/factor analysis (PCA/FA) was the most popular developmental method used by researchers (11 out of 13), while other attempts including regression model [36], summation model, and data-driven approach [37] were also made (Table 2).

### HVI Validation

In the validation of the index, 10 out of 13 used mortality data as the validation variable, while morbidity [38], ambulance callout [33], hospital admissions [39], and emergency department visits and admissions [23] were also used (Table 3). The time span of the used health data started from 1990 and ended in 2015 and most clustered between 2000 and 2005. The duration of the validation data ranged from 1 to 17 years, with an average of 8 years. Three studies used the health data shorter than 3 years, while 62% (8 out of 13) of the studies used datasets of 8 years (the average level) or longer duration. The studies with the longest duration of dataset of 17 years were led by Kim et al. and Krstic et al. [36, 37]. In the studies where the sample size was known, the number of geographical units was in the range of 159 to 4765 (Table 3). The average sample size in studies considering census tract as a geographical unit was 2034.

A variety of mathematical and statistical methods were used to evaluate the performance of HVI (Table 3). The regression model was a common method for index validation (9/13), including Poisson regression, logistic regression, and negative binomial regression. Besides, simple and intuitive approaches, such as the skill scores and scatterplot, were also used. Wolf et al. [33] calculated the skill scores for the dichotomous outcome to assess whether a priori the HVI was a good predictor of high levels of mortality and ambulance callout.

Regarding the results of the validation, most studies showed some increase in the risk of negative health outcomes with a higher HVI. However, the strength of the relationship varied with studies and was not strong.

## Discussion

### General Characteristics of the Included Studies

Although there are increasing studies applying the HVI in heat vulnerability assessment worldwide, few indices have been validated with observed health data, most of which are limited in the USA where SoVI was first proposed and developed by Cutter et al. in 2003 [18]. Benefiting from the abundant census datasets in the USA, numerous studies have developed, improved, and validated the HVI on the basis of the research of Cutter et al. [22, 29, 40]. Therefore, this implies that more validation studies are needed in other parts of the world to have a robust HVI. Seven studies which used census-based unit as the geographical unit of spatial analysis were conducted in the USA, which was also benefited from the open high-resolution census data in their country. Using administrative area “county” as the geographical unit, a vulnerability map at the national level in Korea depicted the distribution of heat-related deaths to help decision-making for disaster resource management and to identify heat risk at spatial scales [36]. Only the study conducted in Shanghai, China, by He et al. [35•] used a spatial resolution of a 500-m grid. With the more common use of remote sensing data, the HVI can provide an assessment at finer spatial resolution and more detailed and accurate information for risk management [35•].

### Factors Associated with HVI Development

According to the selected studies, factors are chosen subjectively by the authors, based on a literature review and intuitive understanding of human-environment interaction [31]. The factors were diverse, and there is no generic factor set for heat vulnerability assessment [41]. For example, Brooks et al. [31] identified 11 key factors that exhibited a strong relationship with mortality associated with climate-related disasters: (1) population with access to sanitation, (2) literacy rate, 15–24-year-olds, (3) maternal mortality, (4) literacy rate, over 15 years, (5) calorific intake, (6) voice and accountability, (7) civil liberties, (8) political rights, (9) government effectiveness, (10) literacy ratio (female to male), and (11) life expectancy at birth, which can be divided into three broad categories—health status, governance, and education. Cutter et al. [18] listed the population, environmental, and social factors influencing human vulnerability to heat, including age, gender, race, socioeconomic status, special needs populations, quality of human settlements, and the built environment. Moreover, using a combination of a meta-analysis with a meta-knowledge approach, Romero-Lankao et al. [42] found that the urban vulnerability to temperature-related hazards has mostly been examined using 13 factors: hazard magnitude (i.e., temperature level), population density, age, gender, pre-existing medical conditions, education, income, poverty, minority status, acclimatization, and access to home amenities. Based on the amount of empirical evidence and the degree of agreement, these drivers account for 66% of the total tallies in vulnerability determinants. In this review, we found that the factors for demographic characteristics, socioeconomic conditions, and the built environment were frequently used in the development of HVI, which is consistent with the above-mentioned findings. However, we also found that the factors associated with hazard exposure and underlying health were chosen less and no factor regarding governance was used in the selected studies. In the following sections, the main factors used in HVI development would be discussion in detail.

### Demographic Characteristics

Demographic characteristics could influence the sensitivity of the populations suffered from hazards, including race, age, and education level. Race contributes to heat vulnerability through the lack of access to resources, cultural differences, and the social, economic, and political marginalization that is often associated with racial disparities [18]. Reid et al. [38] employed the percentage of the population of a race other than white into the HVI development, to express the increased vulnerability caused by race. Age has been demonstrated to be an important factor in the relationship between temperature and health [43]. Owing to the poor thermoregulation ability of the elderly and children, the adverse effect of heat is larger in these groups. Furthermore, elderly individuals often have underlying diseases, and children do not spontaneously adapt their behavior and activities to heat, which may aggravate their sensitivity.

### Socioeconomic Conditions

Socioeconomic conditions reflect if people afflicted by a disaster have access to enough resources, information, and support. People with limited social cohesion have less support when an extreme heat event occurs, which has been identified as a risk factor for heat vulnerability [44, 45]. Living alone was the most frequently employed factor in the development of HVI to reflect the lacking social cohesion and often combined with the elderly to represent the synergy effect from social cohesion and age [23, 46, 47••]. Economic status is selected in the development of the HVI because it reflects the response ability of individuals or households to mitigate the adverse health impact due to heat, which is an essential component of vulnerability. Income and the percentage of the population below the poverty lien are often used to represent the economic status directly, while Wolf et al. [27] employed the percentage of population receiving any kind of social benefit as an indirect factor of it. However, employment is a proxy of economic status and can be used when economic data are unavailable [23].

### Built Environment

Built environment factors play an important role in mitigation or aggravation of heat vulnerability of populations. Landscape often refers to the compositional and configurational patterns of land use and land cover, which has been shown to have a significant effect on climate through various pathways that modulate LST, thereby improving psychosocial health and heat vulnerability [48–51]. Several studies employed vegetation coverage as a proxy of environmental mitigation to heat exposure in the HVI development [35, 39, 52]. Moreover, air conditioning has been proposed as one of the key factors explaining reductions of vulnerability and represents an effective heat adaptation strategy [53••]. Percentage of households with air conditioning (or similar factor) was employed in the HVI development as an adaptation measure to improve the environmental temperature in several studies [38, 39, 46, 54].

### Hazard Exposure

Hazard exposure reflects the exposure of human populations to heat, including intensity (e.g., daily maximum, minimum, and mean temperature), duration (e.g., hot days that are defined as the days with maximum temperature over a certain threshold in summer), variance (e.g., temperature range), and frequency of the hazard. Romero-Lankao et al. [42] found that most studies showed a positive correlation between hazard magnitude and mortality and concluded that the hazard magnitude was the only determinant that has been extensively studied and shows a high level of agreement in its effects across different studies. In our review, only five studies employed the factors of hazard exposure. The unbalanced weights in hazard exposure and social characteristics indicate that the current HVIs have placed particular emphasis on social vulnerability and are similar to the SoVI developed by Cutter et al. [18]. Therefore, more factors associated with hazard exposure need to be employed in HVI development. Land surface temperature (LST) [40, 52] derived from remote sensing and air temperature data [35, 36] of meteorological stations are the main factors used to reflect the level of heat exposure. However, they can be markedly different owing to various complex factors including solar insolation intensity, wind, clouds, shading, sky-view factor, and sensor view angle [55–57]. Hulley et al. argued that LST and air temperature should be included together to fully describe the effects of urban heat because health impacts are tied to both air temperature (through convective processes) and LST (through radiative emission) in 2019 [58•]. In addition, considering the interaction between temperature and humidity, the term “humidex” was used by Krstic et al. [37] to estimate the apparent temperature, which could be a reference for more developments of the HVI.

### Underlying Health

The underlying health condition can greatly affect the sensitivity of an individual to physical and mental health stressors when a heat attack occurs. The percentage of the population with long-term limiting illness (e.g., diabetes and disability) was often used to reflect the negative pre-existing health condition that can increase the vulnerability to heat [23, 33, 38, 39, 46, 59]. On the other hand, medical resources play an important role in providing medical support to the population at risk and maintaining residents’ health. He et al. [35•] calculated the cost of walking time to the nearest medical sites to be used as a proxy for the availability of medical resources in the study area. However, more attention should be given to the insufficient employment of health factors in the HVI in the future.

### Future Considerations

We found that no study used a factor related to governance. Institutional capacity and governance capacity are fundamental to emergency response and disaster management. Zhang et al. adopted a factor of governance in their HVI to measure urban vulnerability to heat wave in Beijing, China [25, 60•]. In addition, knowledge, attitude, and practice (KAP) could greatly influence the adaptive capacity of individual. However, this type of factor, such as city awareness and commitment to adapt, has only been explicitly used in a limited number of studies [61, 62]. Therefore, we argued that the use of factors reflecting governance and awareness should be emphasized in the development of the HVI.

### Methods for HVI Development

Regarding the method used for HVI development, PCA has been commonly used [38, 63]. PCA is a data transformation technique used to reduce multidimensional datasets to a lower number of dimensions for further analysis, which belongs to an inductive design [47••]. Using uncertainty and sensitivity analysis, Tate [32] assessed the methods used in the most common social vulnerability index designs (i.e., deductive, hierarchical, and inductive). Compared with other models, this study found that the inductive design such as PCA was the most precise and was most sensitive to the indicator set and scale of analysis. However, after implementing PCA, principal components are not as readable and interpretable as original features. A variety of studies have developed unsupervised HVIs [64], including some of the selected studies in our review. Given that unsupervised HVI metrics are sensitive to input variables, Conlon et al. [47••] developed a supervised HVI using mortality data to select the variables used for the index calculation, which insured that the index values reflected an indication of vulnerability for this region. In addition, Krstic et al. [37] proposed a data-driven Heat Exposure Integrated Deprivation Index approach to be an effective alternative or to supplement conventional approaches. Using this approach, larger areas (and therefore larger populations) are identified as being at moderate risk. However, index development is context dependent, and there is no single methodology that allows a standardized assessment and produces comparable results [65]. Romero-Lankao et al. conducted an analysis of 54 papers on urban vulnerability to temperature-related hazards. They argued that it was possible to identify common patterns of vulnerability across urban centers and research paradigms, and these commonalities hold the potential for the development of a common set of tools to enhance response capacity within multiple contexts [42]. Hence, improvement of the current methods and the development of innovative methods are urgently needed.

### Validation Variables

For the validation variables, mortality data were much more frequently used. Compared with other health outcomes, mortality reflects a more direct influence on human health and shows less bias in the exposure-response relationship between heat and health. Longer duration and more locations for the validation factors will be helpful for a more precise evaluation of the HVI. However, because of the privacy of health data, it is still quite difficult to access high-quality data with a longer time span, large sample size, and high geographical resolution, which are challenges for the validation work. We suggest making health data more open and available on the premise of data security.

### Performance of HVI

According to the selected articles in this review, a higher value of the HVI indicates an increased heat risk, but we found that the HVI might not be strongly associated with heat-related health outcomes. Based on the method proposed by Reid et al. [66], Mallen et al. [46] developed an HVI using PCA at the census tract level in Dallas, TX, USA. The bivariate spatial regressions resulted in a coefficient of determination (denoted *R*^2^) of 0.03, which indicated very little correlation between the total deaths and the HVI score. Conlon et al. [47••] developed HVIs using unsupervised and supervised PCA. When validated them using the proportion of deaths occurring on an extreme heat day, the unsupervised and supervised HVIs resulted in very low *R*^2^, potentially indicating that the indices were inadequately capturing spatial variations in heat risk across the study area. These facts implied that the index should be used with caution for identifying the vulnerable areas where heat-related mortality is likely to increase. To further enhance the reliability of HVI, the duration and locations of validation data should be strengthened to verify the reliability of HVI.

## Conclusion

The HVI is a relatively simple and direct method of transmitting information on heat vulnerability to stakeholders. It can be used to highlight the areas where the population is at a high risk of heat, understand the heat vulnerability distribution, provide evidence-based support for decision-making, and even project the scenario of future vulnerability. In the present review, we have examined the key factors, methods, and spatial units used in the development and validation of HVI in detail to further discuss the underlying limitations that are helpful for future studies. The factors related to demographic characteristics, socioeconomic conditions, and the built environment have been widely used in the development of the HVI, while more factors associated with hazard exposure, underlying health, governance, and protection awareness should be integrated to develop a comprehensive index of heat vulnerability. In fact, various factors affected the HVI, and the omission of key issues may result in misleading conclusions. Considering the key factors as fully as possible enables to reflect the real situation of heat vulnerability.

However, we found that the HVI may not be strongly associated with heat-related health outcomes, and further validation of the HVI should be conducted in various locations to verify the reliability of index. Moreover, the duration and location of validation data is of great importance to enhance the reliability of HVI.

## Supplementary Information

ESM 1 (DOCX 14 kb)

ESM 2 (DOCX 19 kb)

ESM 3 (DOCX 19 kb)
