# Supplementary material for: A Systematic Review of the Development and Validation of the Heat Vulnerability Index: Major Factors, Methods, and Spatial Units
Source: Curr Clim Change Rep. 2021 Apr 27;7(3):87–97. doi: 10.1007/s40641-021-00173-3 (PMC8531084; doi:10.1007/s40641-021-00173-3)
Supplement: Supplementary file 1 — (DOCX 14 kb) [file 40641_2021_173_MOESM1_ESM.docx]

Supplementary Material 1. Search strategy of articles

The searches were performed in five on-line bibliographic databases including PubMed, Web of Science, Science Direct, China National Knowledge Infrastructure and Wanfang Data between January 2010 and October 2020 and considered either “all fields” (including bibliography references) or only “title-keywords-abstract” according to the database query form, and limited to the type “journal article” and language “English and Chinese”. The logical structure of the queries was based on the following formula:

((heat OR heatwave* OR ("high temperature") OR thermal) AND (vulnerability OR risk) AND (index OR indices) AND (assess OR assessment OR evaluation) AND health)

The character * being the classical symbol for regular expressions, corresponding to any character or group of characters. Quotation marks can be used to specify terms which must appear next to each other.
