# Supplementary material for: A Systematic Review of the Development and Validation of the Heat Vulnerability Index: Major Factors, Methods, and Spatial Units
Source: Curr Clim Change Rep. 2021 Apr 27;7(3):87–97. doi: 10.1007/s40641-021-00173-3 (PMC8531084; doi:10.1007/s40641-021-00173-3)
Supplement: Supplementary file 2 — (DOCX 19 kb) [file 40641_2021_173_MOESM2_ESM.docx]

Supplementary Material 2. The list of excluded 33 studies.

1. Aubrecht, C. and D. Özceylan (2013). "Identification of heat risk patterns in the U.S. National Capital Region by integrating heat stress and related vulnerability." Environ Int 56: 65-77.
2. Azhar, G., S. Saha, P. Ganguly, D. Mavalankar and J. Madrigano (2017). "Heat Wave Vulnerability Mapping for India." Int J Environ Res Public Health 14(4).
3. Bai, L., A. Woodward, Cirendunzhu and Q. Liu (2016). "County-level heat vulnerability of urban and rural residents in Tibet, China." Environ Health 15: 3.
4. Bradford, K., L. Abrahams, M. Hegglin and K. Klima (2015). "A Heat Vulnerability Index and Adaptation Solutions for Pittsburgh, Pennsylvania." Environ Sci Technol 49(19): 11303-11311.
5. Cai, Z., Y. Tang, K. Chen and G. Han (2019). "Assessing the Heat Vulnerability of Different Local Climate Zones in the Old Areas of a Chinese Megacity." Sustainability 11(7).
6. Chow, W., W.-C. Chuang and P. Gober (2012). "Vulnerability to Extreme Heat in Metropolitan Phoenix: Spatial, Temporal, and Demographic Dimensions." The Professional Geographer 64: 286-302.
7. Christenson, M., S. D. Geiger, J. Phillips, B. Anderson, G. Losurdo and H. A. Anderson (2017). "Heat Vulnerability Index Mapping for Milwaukee and Wisconsin." J Public Health Manag Pract 23(4): 396-403.
8. Depietri, Y., T. Welle and F. G. Renaud (2013). "Social vulnerability assessment of the Cologne urban area (Germany) to heat waves: links to ecosystem services." International Journal of Disaster Risk Reduction 6: 98-117.
9. El-Zein, A. and F. N. Tonmoy (2015). "Assessment of vulnerability to climate change using a multi-criteria outranking approach with application to heat stress in Sydney." Ecological Indicators 48: 207-217.
10. Eum, J.-H., K. Kim, E.-H. Jung and P. Rho (2018). "Evaluation and Utilization of Thermal Environment Associated with Policy: A Case Study of Daegu Metropolitan City in South Korea." Sustainability 10(4).
11. Guo, X., G. Huang, P. Jia and J. Wu (2019). "Estimating Fine-Scale Heat Vulnerability in Beijing Through Two Approaches: Spatial Patterns, Similarities, and Divergence." Remote Sensing 11(20).
12. Hammer, J., D. G. Ruggieri, C. Thomas and J. Caum (2020). "Local Extreme Heat Planning: an Interactive Tool to Examine a Heat Vulnerability Index for Philadelphia, Pennsylvania." J Urban Health 97(4): 519-528.
13. Ho, H. C., A. Knudby, G. Chi, M. Aminipouri and D. Yuk-FoLai (2018). "Spatiotemporal analysis of regional socio-economic vulnerability change associated with heat risks in Canada." Applied geography (Sevenoaks, England) 95: 61-70.
14. Hulley, G., S. Shivers, E. Wetherley and R. Cudd (2019). "New ECOSTRESS and MODIS Land Surface Temperature Data Reveal Fine-Scale Heat Vulnerability in Cities: A Case Study for Los Angeles County, California." Remote Sensing 11(18).
15. Inostroza, L., M. Palme and F. de la Barrera (2016). "A Heat Vulnerability Index: Spatial Patterns of Exposure, Sensitivity and Adaptive Capacity for Santiago de Chile." PLoS One 11(9): e0162464.
16. Jänicke, B., A. Holtmann, K. R. Kim, M. Kang, U. Fehrenbach and D. Scherer (2019). "Quantification and evaluation of intra-urban heat-stress variability in Seoul, Korea." Int J Biometeorol 63(1): 1-12.
17. Johnson, D., J. Webber, K. Ravichandra, V. Lulla and A. Stanforth (2014). "Spatiotemporal variations in heat-related health risk in three Midwestern US cities between 1990 and 2010." Geocarto International 29.
18. Loughnan, M., N. Nicholls and N. Tapper (2012). "Mapping Heat Health Risks in Urban Areas." International Journal of Population Research 2012.
19. Méndez-Lázaro, P., F. E. Muller-Karger, D. Otis, M. J. McCarthy and E. Rodríguez (2018). "A heat vulnerability index to improve urban public health management in San Juan, Puerto Rico." Int J Biometeorol 62(5): 709-722.
20. Mushore, T. D., O. Mutanga, J. Odindi and T. Dube (2018). "Determining extreme heat vulnerability of Harare Metropolitan City using multispectral remote sensing and socio-economic data." Journal of Spatial Science 63(1): 173-191.
21. Peng, Y., Y. Chen and X. Lin (2019). "Vulnerability assessment of heat wave in Qingyuan City." Journal of Qingyuan Polytechnic 12(1): 19-23.
22. Rasanen, A., K. Heikkinen, N. Piila and S. Juhola (2019). "Zoning and weighting in urban heat island vulnerability and risk mapping in Helsinki, Finland." Regional Environmental Change 19(5): 1481-1493.
23. Reid, C. E., J. K. Mann, R. Alfasso, P. B. English, G. C. King, R. A. Lincoln, H. G. Margolis, D. J. Rubado, J. E. Sabato, N. L. West, B. Woods, K. M. Navarro and J. R. Balmes (2012). "Evaluation of a Heat Vulnerability Index on Abnormally Hot Days: An Environmental Public Health Tracking Study." Environmental Health Perspectives 120(5): 715-720.
24. Rinner, C., D. Patychuk, K. Bassil, S. Nasr, S. Gower and M. Campbell (2010). "The Role of Maps in Neighborhood-level Heat Vulnerability Assessment for the City of Toronto." Cartography and Geographic Information Science 37(1): 31-44.
25. Tapia, C., B. Abajo, E. Feliu, M. Mendizabal, J. Antonio Martinez, J. German Fernandez, T. Laburu and A. Lejarazu (2017). "Profiling urban vulnerabilities to climate change: An indicator-based vulnerability assessment for European cities." Ecological Indicators 78: 142-155.
26. Tran, D. N., V. Q. Doan, V. T. Nguyen, A. Khan, P. K. Thai, H. Cunrui, C. Chu, E. Schak and D. Phung (2020). "Spatial patterns of health vulnerability to heatwaves in Vietnam." Int J Biometeorol 64(5): 863-872.
27. Walton, Z. L., N. C. Poudyal, J. Hepinstall, C. Johnson Gaither and B. B. Boley (2015). "Exploring the role of forest resources in reducing community vulnerability to the heat effects of climate change." Forest Policy and Economics 71: 94-102.
28. Wang, S. (2019). "Evaluation of Urban High Temperature Vulnerability in Zhangzhou Based on Multi-source Spatial Data." Taiwan Agricultural Research(6): 69-75.
29. Zhang, M., Z. Liu and M. P. van Dijk (2019). "Measuring urban vulnerability to climate change using an integrated approach, assessing climate risks in Beijing." Peerj 7.
30. Zhang, W., P. McManus and E. Duncan (2018). "A Raster-Based Subdividing Indicator to Map Urban Heat Vulnerability: A Case Study in Sydney, Australia." International Journal of Environmental Research and Public Health 15(11).
31. Zheng, M., J. Zhang, L. Shi, D. Zhang, T. P. Pangali Sharma and F. A. Prodhan (2020). "Mapping Heat-Related Risks in Northern Jiangxi Province of China Based on Two Spatial Assessment Frameworks Approaches." Int J Environ Res Public Health 17(18).
32. Zhu, Q., T. Liu, H. Lin, J. Xiao, Y. Luo, W. Zeng, S. Zeng, Y. Wei, C. Chu, S. Baum, Y. Du and W. Ma (2014). "The spatial distribution of health vulnerability to heat waves in Guangdong Province, China." Global Health Action 7.
33. Zuhra, S. S., A. B. Tabinda and A. Yasar (2019). "Appraisal of the heat vulnerability index in Punjab: a case study of spatial pattern for exposure, sensitivity, and adaptive capacity in megacity Lahore, Pakistan." Int J Biometeorol 63(12): 1669-1682.
