# Supplementary material for: A Systematic Review of the Development and Validation of the Heat Vulnerability Index: Major Factors, Methods, and Spatial Units
Source: Curr Clim Change Rep. 2021 Apr 27;7(3):87–97. doi: 10.1007/s40641-021-00173-3 (PMC8531084; doi:10.1007/s40641-021-00173-3)
Supplement: Supplementary file 3 — (DOCX 19 kb) [file 40641_2021_173_MOESM3_ESM.docx]

Supplementary Material 3. Detailed inventory of factors used in HVI development in the selected studies.

| ID | Hazard Exposure | Demographic Characteristics | Socioeconomic Conditions | Built Environment | Underlying Health |
| --- | --- | --- | --- | --- | --- |
| 1 | **Exposure intensity:** Land surface temperature | **Age:** Females age 65 and up; Males age 65 and up; **Race and/or ethnicity:** White population; Asian population; Other race population; Hispanic population; Black population;  **Education:** Population 25 and older with less than high school education; Population 25 and older with a high school education;  **Family structure:** Females head of household; | **Economic status:** Mean family income in 1989; Per capita income in 1989; Mean household income in 1989; **Social cohesion:** Males age 65 and up living alone; Females age 65 and up living alone; Population age 65 and older in group living; | **Landscape:** Normalized difference built-up index; Normalized difference vegetation index; |  |
| 2 |  | **Age:** Percentage of population > 65 years of age  **Race and/or Ethnicity:** Percentage of population of a race other than white; **Education:** Percentage of population with less than a high school diploma; | **Economic status:** Percentage of population below the poverty line; **Social cohesion:** Percentage of population that live alone; Percentage of population > 65 years of age that live alone; | **Landscape:** Percentage of nongreen space; **Adaptation measures:** Percentage of homes without central air conditioning; Percentage of homes with no air conditioning of any kind; | **Health condition:** Percentage of population diagnosed with diabetes; |
| 3 |  | **Age:** ≥ 65 years of age;  **Race and/or Ethnicity:** Ethnic minority;  **Immigrant:** Latino immigrant; **Education:** No High School diploma; | **Economic status:** < Poverty line; **Social cohesion:** ≥ 65 years of age × living alone; Living alone; | **Landscape:** Unvegetated area(MEAN); Unvegetated area(SD)  **Adaptation measures:** No central AC/cooler; |  |
| 4 |  | **Age:** Population above 65 years old;  **Race and/or Ethnicity:** Ethnic group other than “white British” **Family structure:** Single pensioner households; **Population size:** Population density; | **Economic status:** Receiving any kind of social benefit;  **Access to housing:** Households in rented tenure; Households in a flat; | **Adaptation measures:** Households without central heating; | **Health condition:** Population with long-term limiting illness (Population with self-reported health status “not good”); |
| 5 |  | **Age:** Percent population ≥65 years of age;  **Race and/or Ethnicity:** Percent population of a race other than white; **Education:**  Percent population ≥25 years of age and does not hold a high school degree; | **Economic status:** Percent population living in poverty; **Social cohesion:** Percent population living alone; Percent population ≥65 years of age and living alone; | **Landscape:** Percent county with land use/land cover described as urban | **Health condition:** Percent population ever diagnosed with diabetes; |
| 6 |  | **Age:** Age > 65 years;  **Race and/or Ethnicity:** Race/ethnicity other than non-Hispanic white;  **Education:** Less than high school diploma; | **Economic status:** Below poverty line;  **Social cohesion:** Living alone; Elderly living alone; | **Landscape:** Low vegetation cover;  **Adaptation measures:** No central AC; No AC of any kind | **Health condition:** Diabetes; |
| 7 | **Exposure intensity:** Surface temperature | **Age:** Age (0–5 years & 65+ years);  **Race and/or Ethnicity:** Ethnicity (black, non-Hispanic white, Hispanic, other); **Population size:** Population density; | **Social cohesion:** Socially isolated; **Access to housing:** Renters status; | **Landscape:** Impervious surface or lack of vegetative cover; |  |
| 8 | **Exposure duration:** Tropical nights; Heatwave days | **Occupation:** Agricultural workers | **Social cohesion:** elderly living alone |  |  |
| 9 | **Exposure intensity:** Maximum Humidex | **Age:** Senior density | **Socioeconomic status:** Vancouver Area Neighborhood Deprivation Index (VANDIX) | **Urban form:** Road Density |  |
| 10 |  | **Age:** Percentage population over 65 years of age;  **Race and/or Ethnicity:** Percentage population that is Hispanic; Percentage population that is Black;  **Immigrant:** Percentage population that is foreign born;  **Language:** Percentage population who speak English less than ‘very well’; | **Economic status:** Percentage population with income below poverty level; **Social cohesion:** Percentage population over 65 years of age and living alone;  **Employment:** Percentage population (18-64 years) that are unemployed; | **Landscape:** Percentage land with high building intensity areas; Percentage land that consists of open undeveloped areas;  **Building characteristics:** Percentage houses built before 1980; Density of housing units per square mile; | **Health condition:** Percentage population (18-64 years) that has a disability; |
| 11 | **Exposure duration:** Heat wave duration;  **Exposure frequency:** Heat wave frequency; **Exposure intensity:** Heat wave intensity; |  | **Economic status:** Night-time light (NTL) value | **Landscape:** Vegetation coverage; **Urban form:** Density of roads; | **Medical resources:** Availability of medical resources; |
| 12 |  | **Age:** Over age 65;  **Race and/or Ethnicity:** Race other than white; **Education:** Less than high school education; | **Economic status:** Living below poverty line; **Social cohesion:** Living alone; Over age 65 and living alone; | **Landscape:** No green space; **Adaptation measures:** No AC access; NO full AC access | **Health condition:** Diabetes prevalence; |
| 13 |  | **Age:** Over age 65;  **Race and/or Ethnicity:** Minority  **Education:** Less than HS education | **Economic status:** Under poverty level  **Social cohesion:** Living alone; Over age 65, living alone | **Landscape:** Distance to water; Nonvegetated, including water OR Nontrees OR Impervious surface OR Nontree canopy |  |
